# A Case of Moderately Severe COVID-19 in a Healthcare Worker in Russia: Virus Isolation and Full Genome Sequencing

**DOI:** 10.7759/cureus.13733

**Published:** 2021-03-06

**Authors:** Alexey M Shchetinin, Elena V Tsyganova, Denis N Protsenko, Andrey G Botikov, Vladimir Gushchin

**Affiliations:** 1 Pathogenic Microorganisms Variability Laboratory, N.F. Gamaleya National Research Centre for Epidemiology and Microbiology, Ministry of Health of the Russian Federation, Moscow, RUS; 2 Department of Clinical Science, Moscow City Centre for AIDS Prevention and Treatment, Moscow, RUS; 3 Anesthesiology, Moscow City Clinical Hospital № 40, Moscow, RUS; 4 Anesthesiology and Reanimatology, Pirogov Medical University, Moscow, RUS; 5 Russian State Collection of Viruses, N.F. Gamaleya National Research Centre for Epidemiology and Microbiology, Ministry of Health of the Russian Federation, Moscow, RUS

**Keywords:** ngs, covid 19, health-care worker, virus isolation, sars-cov-2, clinical case

## Abstract

The coronavirus disease 2019 (COVID-19) pandemic is probably the most studied one in history from both clinical and molecular-epidemiological perspectives. Nonetheless, data on the correlation between severe acute respiratory syndrome coronavirus 2 (SARS-CoV-2) viral genotypes and COVID-19 symptoms caused by them are still scarce. In this report, we present a moderately severe COVID-19 case in a healthcare worker in Moscow, Russia, supplemented with the data on its causative agent's phenotype regarding *in vitro* and full-genome characterization. The 44-year-old male healthcare worker who had frequent professional contacts with COVID-19 patients was hospitalized with a viral pneumonia diagnosis and soon started to exhibit fever, dry paroxysmal cough, loss of smell, and typical ground-glass opacities found in both lungs on chest CT scans. The COVID-19 diagnosis was verified by real-time quantitative polymerase chain reaction (qRT-PCR), immunochromatography, and immunochemiluminescent assays. The patient was treated with hydroxychloroquine, azithromycin, paracetamol, and enoxaparin, leading to his recovery after two weeks from the disease onset. The virus was successfully isolated from the nasopharyngeal swab sample taken on the fifth day of the disease onset using the Vero E6 cell line and exhibited a pronounced cytopathic effect (CPE) with a viral titer reaching 10^6^ TCID_50_/ml in the cell culture medium. The full genome sequence of the viral isolate was obtained and 8 nucleotide and 5 amino acid mutations compared to the Wuhan-Hu-1 reference genome were identified. Viral isolate belonged to GR / 20B / B.1.1 genetic lineage (GISAID, Nextstrain, Pangolin nomenclatures, respectively) - the most prevalent genotype found in Russia to date.

## Introduction

For more than nine months during the period of active severe acute respiratory syndrome coronavirus 2 (SARS-CoV-2) circulation, a large amount of data was accumulated on coronavirus disease 2019 (COVID-19) clinical presentations [1,2], as well as molecular epidemiological data representing the high activity of circulating strains [3-5]. The emergence of SARS-CoV-2 and the pandemic it caused is possibly the most studied one in history from both clinical and molecular-epidemiological perspectives. Nonetheless, data on the correlation between SARS-CoV-2 viral genotypes and COVID-19 symptoms caused by them are still scarce. In this report, we present a COVID-19 case study of a healthcare worker in Moscow, Russia, supplemented with the data on its causative agent phenotype regarding *in vitro* and full-genome characterization.

## Case presentation

The patient, a 44-year-old male, was hospitalized on 30.03.2020 (the second day after the onset of the disease) with viral pneumonia. Frequent professional contacts with COVID-19 patients were described in his medical history. Upon admission to the hospital, the patient’s condition was assessed as moderate with complaints of weakness, fatigue, and fever. Since the first day of the disease onset (29.03.2020), the patient had exhibited moderate fatigue and chills, but his temperature had not been measured and he had taken paracetamol to alleviate the symptoms. On the third day, a dry paroxysmal cough appeared, and on the fourth day, the patient noted the loss of smell. The fever persisted for 10 days with body temperature ranging from 37.4 to 39.2 °С. The patient was on eprosartan + hydrochlorothiazide 600 mg, atorvastatin 40 mg, and nebivolol 2.5 mg for his arterial hypertension.

On examination, skin and sclera were of normal color, with no rash. The mucous membrane of the oropharynx was moderately hyperemic, tonsils were not enlarged, with no plaque. Peripheral lymph nodes were not enlarged and were painless on palpation. Breathing was not impaired; the frequency of respiratory movement was 18 per minute, with SpO2 of 96%. Heart sounds were rhythmic, without abnormal sounds, heart rate was 78 beats per minute, and blood pressure was 130/90 mmHg. The abdomen was soft and painless upon palpation, and the liver was at the costal margin. The consciousness was clear, with no focal or meningeal symptoms. Urination was not impaired, and there was no diarrhea.

Blood tests and biochemical results in dynamic are presented in Tables 1, 2, 3. At the onset of the disease, leukopenia and a slight increase in monocytes percentage were noted. An increase in acute phase indicators such as C-reactive protein, erythrocyte sedimentation rate (ESR), and fibrinogen was observed. Of particular interest was a fairly high level of C-reactive protein, which is characteristic of COVID-19, while the procalcitonin test was negative. Minor changes in the biochemical analysis of blood were clinically insignificant and, possibly, reflected the fact that the patient was taking certain medications. Ferritin and interleukin-6 levels were not assessed during this study.

**Table 1:** Complete blood count results Values that exceed the reference range are marked with an asterisk

Variable	Value		Reference range		Units
	03.04.2020	10.04.2020			
White blood cells	3.0	6.2	4.0 – 9.0		10Е9/L
Neutrophils	1.8 / 58.7%	4.6 / 74%	1.7 – 7.7	42 – 85%	10Е9/L
Lymphocytes	0.6 / 20.1%	0.9 / 13.7%	0.4 – 4.4	11 – 49%	10Е9/L
Monocytes*	0.5 / 17.1%*	0.5 / 8.5%	0.0 – 0.8	0 – 11%	10Е9/L
Eosinophils	0.1 / 2.4%	0.1 / 2%	0.0 – 0.6	0 – 6%	10Е9/L
Basophils	0.1 / 1.7%	0.1 / 1.8%	0.0 – 0.2	0 – 2%	10Е9/L
Red blood cells	4.66	5.01	3.8 – 5.3		10Е12/L
Hemoglobin	141	146	110 – 170		g/L
Hematocrit	40.8	43.4	36.0 – 56.0		%
Mean corpuscular volume	87.6	86.6	80.0 – 100		fL
Mean corpuscular hemoglobin	30.3	29.1	28.0 – 36.0		pg
Mean corpuscular hemoglobin concentration	346	336	310 – 370		g/L
Red cell distribution width - coefficient of variation	14.3	14.2	10.0 – 16.5		%CV
Red cell distribution width - standard deviation	50.1	49.2	35 – 60		fL
Platelets	227	361	120 – 380		10E9/L
Procalcitonin	0.15	0.24	0.10 – 1.0		%
Mean platelet volume	6.6	6.6	5.0 – 10.0		fL
Platelet distribution width	17.1	17.9	12.0 – 18.0		%
Erythrocyte sedimentation rate*	14	27*	Men under 50 years of age: 0 – 15 mm/hour. Women under 50 years of age: 0 – 20 mm/hour. After 50 years: up to 30 mm/hour		

**Table 2:** Coagulation testing results Values that exceed the reference range are marked with an asterisk

Variable	Value		Reference range	Units
	03.04.2020	10.04.2020		
Partial thromboplastin time	35.8	33	25.1 – 36.5	Seconds
Ratio (R)*	1.28*	1.18	0.8 – 1.2	-
Prothrombin time*	13.6*	15.2*	9.4 – 12.5	Seconds
Prothrombin index*	80	68*	70 – 140	%
International normalized ratio (INR)	1.18	1.33	0.9 – 1.2	-
Thrombin time	15.2	N/A	10.3 – 16.6	Seconds
Fibrinogen*	3.96*	5.24*	2.00 – 3.93	g/L
D-dimer	96	106	0 – 230	µg/L

**Table 3:** Blood chemistry test results Values that exceed the reference range are marked with an asterisk

Variable	Value			Reference range	Units
	30.03.2020	03.04.2020	10.04.2020		
Glucose	5.9	5.48	5.6	4.20 – 6.40	mmol/L
Cholesterol	4.88	2.09	2.6	0.00 – 6.20	mmol/L
Triglycerides	1.88	1.62	1.75	0.00 – 2.30	mmol/L
High-density lipoprotein*	0.6*	0.65*	0.5*	1.04 – 1.55	mmol/L
Low-density lipoprotein	1.68	1.91	1.77	0.00 – 4.12	mmol/L
Bilirubin total	16.1	7.9	12.5	0.0 – 17.0	µmol/L
Bilirubin direct	3.1	2.3	2.9	0.0 – 4.3	µmol/L
Protein total	71	67.0	68.8	64.0 – 83.0	g/L
Creatinine	83	93	87.81	62 – 98	µmol/L
Urea	4.89	3.83	4.54	1.70 – 8.30	µmol/L
Uric acid*	614*	400*	543.9*	142 – 339	µmol/L
Alanine aminotransferase*	78*	52.5*	54.6*	0.0 – 31.0	U/L
Aspartate aminotransferase*	64*	38.9*	44.7*	0.0 – 31.0	U/L
Amylase	81	79	74.4	28 – 100	U/L
Gamma-glutamyltransferase*	129*	58.2*	N/A	7.0 – 32.0	U/L
Lactate dehydrogenase	288	480	270.29	230 – 460	U/L
Alkaline phosphatase	N/A	272	99.09	98 – 279	U/L
Creatine kinase, total*	N/A	227*	141.17	24 – 170	U/L
Serum iron	N/A	N/A	15.08	9 – 31	µmol/L
Unsaturated iron-binding capacity	N/A	N/A	30.2	12.5 – 55.5	µmol/L
Total iron-binding capacity	N/A	N/A	45.2	44.8 – 80.6	µmol/L
Procalcitonin	N/A	N/A	<0.5	0.00 – 0.2	U/L
Albumin	N/A	42.3	37.5	34.0 – 50.0	g/L
C-reactive protein*	N/A	23.6*	55.95*	0.0 – 5.0	mg/L
Sodium	N/A	139.4	141.35	136.0 – 146.0	mEq/L
Potassium	N/A	3.14	4.1	3.50 – 5.10	mEq/L
Chloride	N/A	94.3	101.4	97.0 – 107.0	mEq/L
Rheumatoid factor	N/A	N/A	9.17	0.0 – 20.0	IU/ml

**Figure 1:**
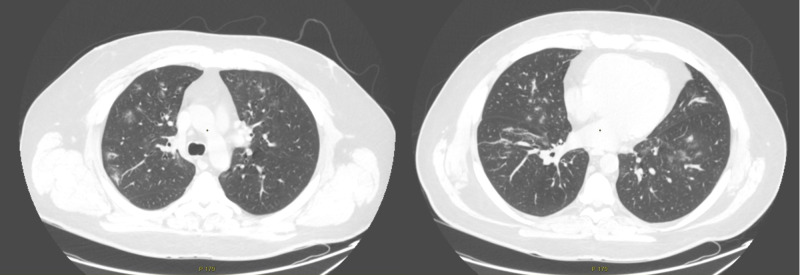
Axial chest CT scans CT scans were taken on the eighth day of the disease onset and revealed up to 45% damage to both lungs with the progression of ground-glass opacities to infiltration CT: computed tomography

Chest CT scans were performed on days two and eight of the disease onset with the following results: 30.03.2020 - up to 25% damage to both lungs with ground-glass opacities; 05.04.2020 - up to 45% damage with the progression of ground-glass opacities to infiltration (Figure 1). Chest X-ray scans were not taken due to the availability of CT scans results.

The COVID-19 diagnosis was verified by polymerase chain reaction (PCR), immunochromatography, and immunochemiluminescent assays (Table 4). SARS-CoV-2 viral RNA was initially detected on 30.03.2020; during this period, no immunoglobulin M (IgM) and immunoglobulin G (IgG) antibodies to the virus were detected using an immunochromatography assay. Viral RNA was detected repeatedly in nasopharyngeal swabs thereafter. Virus persistence was detectable for 10 days since the first positive test. The appearance of specific IgM and IgG antibodies was recorded on the 16th day from the disease onset.

**Table 4:** COVID-19 diagnosis verification results SARS-CoV-2: severe acute respiratory syndrome coronavirus 2; COVID-19: coronavirus disease 2019; IgM: immunoglobulin M; IgG: immunoglobulin G

Date	SARS-CoV-2 RNA	IgM	IgG
30.03.2020	Detected	Negative	Negative
06.04.2020	Detected		
09.04.2020	Detected		
13.04.2020	Not detected	Positive	Positive
14.04.2020	Not detected		
13.05.2020		Positive	Positive
01.06.2020		Positive	Positive

The patient was given detoxification and symptomatic therapy, including hydroxychloroquine (400 mg twice on the first day, 200 mg twice daily during the period covering the second to 10th day), azithromycin (500 mg once per day), paracetamol (up to 4 g per day with a fever above 38.5 °С), and enoxaparin 0.4 ml every 12 hours. On the ninth day of the disease, the patient recovered his sense of smell; his body temperature normalized on the next day, and he was discharged after 14 days from the onset of the disease.

**Figure 2:**
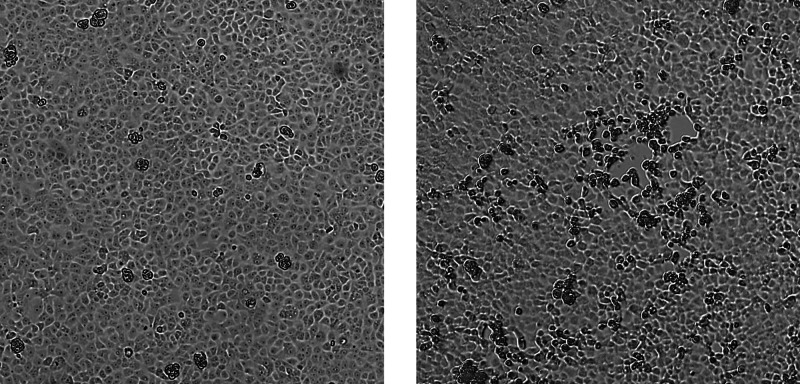
Microphotographs of control cells (left) and cells inoculated with the patient’s nasopharyngeal swab (right) three days post-inoculation The clear cytopathic effect is observed, resulting from cell monolayer degeneration, rounding of the cells, and their detachment from the substrate

**Figure 3:**
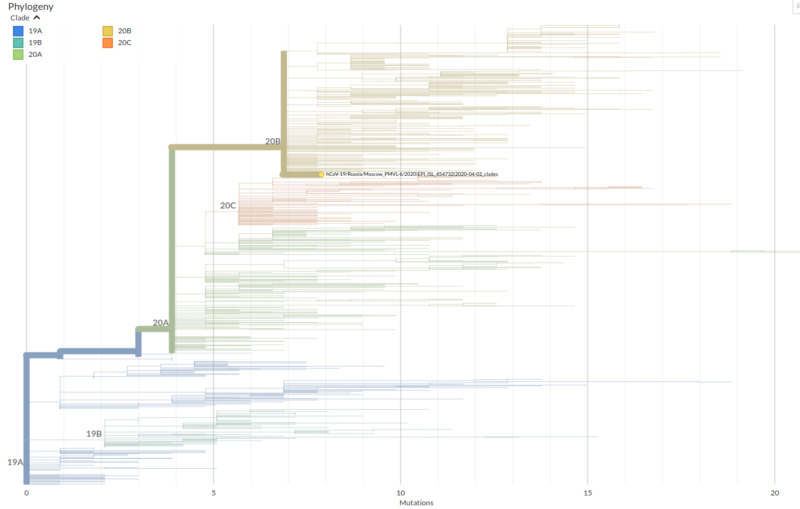
Phylogenetic placement of the studied PMVL-6 isolate The phylogram was provided by the Nextclade tool of the Nextstrain web-service (clades.nextstrain.org). The tree is colored according to clade names; PMVL-6 isolate is highlighted with a yellow dot and full name

A PCR-positive nasopharyngeal swab (taken on the fifth day of the disease) was used to isolate the virus in a Vero E6 cell culture followed by viral genome high-throughput sequencing. The obtained viral isolate exhibited a pronounced cytopathic effect (CPE) on the third day of cell line inoculation (Figure 2) with a viral titer reaching 106 TCID50/ml in the cell culture medium. Successful viral isolation was confirmed using real-time quantitative polymerase chain reaction (qRT-PCR) with WHO and CDC-recommended PCR primers (targeting E, ORF1b, N, and RdRp genes) [6,7]. The DNA library was constructed from a concentrated cell culture medium and sequenced on the Ion S5XL instrument; an almost complete viral genome was deposited into GISAID under the name hCoV-19/Russia/Moscow_PMVL-6/2020 (EPI_ISL_454732). According to the results of the phylogenetic analysis, the studied viral isolate belongs to the GR genetic lineage (GISAID nomenclature), B.1.1 genetic lineage (proposed by Rambaut et al. [8] and implemented in Pangolin software), and 20B clade (Nextstrain nomenclature) (Figure 3), suggesting that the studied isolate is well adapted for spreading among the human population.

Genetic analysis showed 8 nucleotide and 5 amino acid substitutions between PMVL-6 and reference strain Wuhan-Hu-1 (GenBank accession NC_045512.2) in the following regions: 5’-untranslated region (5’-UTR), nsp3 (multi-domain transmembrane protein), nsp12 (RNA-dependant RNA-polymerase, RdRp), nsp13 (helicase), S (spike glycoprotein) and N (nucleocapsid protein) (Table 5).

**Table 5:** Nucleotide and amino acid substitutions between studied PMVL-6 isolate and reference strain Wuhan-Hu-1 Absolute genomic coordinates are presented in accordance with the reference strain; corresponding codons positions of viral proteins are depicted in parenthesis

Genomic region	Viral protein	Nucleotide substitution	Amino acid substitution
5’-UTR		241C>T	
ORF1ab	nsp3 (multi-domain protein)	3037C>T (TTC106TTT)	
	nsp12 (RdRp)	14408C>T (CCT323CTT)	P323L
	nsp13 (helicase)	17206T>C (TAT324CAT)	Y324H
ORF2	S (spike)	23403A>G (GAT614GGT)	D614G
ORF9a	N (nucleocapsid)	28881G>A, 28882G>A (AGG203AAA); 28883G>C (GGA204CGA)	R203K, G204R

## Discussion

We presented in detail the COVID-19 case report of a healthcare worker in Moscow, Russia, supplemented with data on virus propagation in cell culture related to *in vitro* and full-genome sequencing. The clinical picture of COVID-19 in the patient we studied corresponds to the classical description of the course of the new coronavirus infection caused by SARS-CoV-2. The onset of the disease was characterized by intoxication and fever, followed by the appearance of respiratory symptoms and a highly characteristic symptom such as anosmia. Laboratory and diagnostic imaging analysis results revealed changes typical for COVID-19, the severity of which allowed us to exclude the presence of a “cytokine storm” and to assess the course of the disease as moderate in accordance with the WHO classification.

The virus was successfully isolated from a nasopharyngeal swab sample using the Vero E6 cell line and reached a high titer in the culture medium, causing a visible CPE. The obtained viral isolate belongs to the genetic lineage B.1.1 - the most prevalent one in Russia ( >78% sequences). It includes SARS-CoV-2 isolates circulating in 93 different countries (as of 07.10.2020), the most represented of which are the United Kingdom (22,975), USA (3,804), and Australia (1,873). Of particular interest among detected amino acid substitutions is spike D614G mutation, which is present in almost all genomes of viral strains belonging to 20A, 20B, and 20C clades (Nextstrain nomenclature). This substitution is possibly linked with increased SARS-CoV-2 virulence due to its effect on the destabilization of S protein conformation [9], but the level of correlation between this and other detected mutations and moderate severity of the described case remains to be elucidated.

## Conclusions

The case we described is one of the very few globally published case reports of COVID-19 from Russia, and despite its typical clinical picture, it might be of interest to the global community, since it depicts infection with the most common Russian viral genotype to date. The possibility of SARS-CoV-2 nosocomial infections highlights the significance of stringent preventive measures in hospitals and our case highlights this problem, considering the most likely route of the infection in our patient. Continuous characterization of viral genomes and phenotypes *in vitro *is crucial for further improvements in molecular diagnostics, antivirals, and vaccine development.
